# US National Institutes of Health Prioritization of SARS-CoV-2 Variants

**DOI:** 10.3201/eid2905.221646

**Published:** 2023-05

**Authors:** Sam Turner, Arghavan Alisoltani, Debbie Bratt, Liel Cohen-Lavi, Bethany L. Dearlove, Christian Drosten, Will M. Fischer, Ron A.M. Fouchier, Ana Silvia Gonzalez-Reiche, Lukasz Jaroszewski, Zain Khalil, Eric LeGresley, Marc Johnson, Terry C. Jones, Barbara Mühlemann, David O’Connor, Mayya Sedova, Maulik Shukla, James Theiler, Zachary S. Wallace, Hyejin Yoon, Yun Zhang, Harm van Bakel, Marciela M. Degrace, Elodie Ghedin, Adam Godzik, Tomer Hertz, Bette Korber, Jacob Lemieux, Anna M. Niewiadomska, Diane J. Post, Morgane Rolland, Richard Scheuermann, Derek J. Smith

**Affiliations:** University of Cambridge, Cambridge, UK (S. Turner, E. LeGresley, T.C. Jones, B. Mühlemann, D.J. Smith);; University of California Riverside School of Medicine, Riverside, California, USA (A. Alisoltani, L. Jaroszewski, M. Sedova, A. Godzik);; National Institutes of Health, Rockville, Maryland, USA (D. Bratt, M.M. Degrace, E. Ghedin, D.J. Post);; Ben-Gurion University of the Negev, Be’er-Sheva, Israel. (L. Cohen-Lavi, T. Hertz);; Walter Reed Army Institute of Research, Silver Spring, Maryland, USA (B.L. Dearlove, M. Rolland);; Henry M. Jackson Foundation for the Advancement of Military Medicine, Inc., Bethesda, Maryland, USA (B.L. Dearlove, M. Rolland);; Charité–Universitätsmedizin and German Center for Infection Research, Berlin, Germany (C. Drosten, T.C. Jones, B. Mühlemann);; Los Alamos National Laboratory, Los Alamos, New Mexico, USA (W.M. Fischer, J. Theiler, H. Yoon, B. Korber);; Erasmus Medical Center, Rotterdam, the Netherlands (R.A.M. Fouchier);; Icahn School of Medicine at Mount Sinai, New York, New York, USA (A.S. Gonzalez-Reiche, A.Z. Khalil, H. van Bakel);; University of Missouri, Columbia, Missouri, USA (M. Johnson);; University of Wisconsin–Madison, Madison, Wisconsin, USA (D. O’Connor);; Argonne National Laboratory, Lemont, Illinois, USA (M. Shukla);; University of Chicago, Chicago, Illinois, USA (M. Shukla);; J. Craig Venter Institute, La Jolla, California, USA (Z.S. Wallace, Y. Zhang, A.M. Niewiadomska, R. Scheuermann);; University of California, San Diego, California, USA (Z.S. Wallace, R. Scheuermann);; Broad Institute of MIT and Harvard, Boston, Massachusetts, USA (J. Lemieux);; La Jolla Institute for Immunology, La Jolla (R. Scheuermann);; Global Virus Network, Baltimore, Maryland, USA (R. Scheuermann)

**Keywords:** COVID-19, 2019 novel coronavirus disease, coronavirus disease, severe acute respiratory syndrome coronavirus 2, SARS-CoV-2, viruses, respiratory infections, zoonoses, SARS-CoV-2 variants, US National Institutes of Health, US National Institute of Allergy and Infectious Diseases, epidemiological monitoring, pandemic prevention and control, epidemiology, computational biology

## Abstract

Since late 2020, SARS-CoV-2 variants have regularly emerged with competitive and phenotypic differences from previously circulating strains, sometimes with the potential to escape from immunity produced by prior exposure and infection. The Early Detection group is one of the constituent groups of the US National Institutes of Health National Institute of Allergy and Infectious Diseases SARS-CoV-2 Assessment of Viral Evolution program. The group uses bioinformatic methods to monitor the emergence, spread, and potential phenotypic properties of emerging and circulating strains to identify the most relevant variants for experimental groups within the program to phenotypically characterize. Since April 2021, the group has prioritized variants monthly. Prioritization successes include rapidly identifying most major variants of SARS-CoV-2 and providing experimental groups within the National Institutes of Health program easy access to regularly updated information on the recent evolution and epidemiology of SARS-CoV-2 that can be used to guide phenotypic investigations.

As part of the US National Institutes of Health (NIH) National Institute of Allergy and Infectious Diseases (NIAID) SARS-CoV-2 Assessment of Viral Evolution (SAVE) effort to combat the SARS-CoV-2 pandemic, the NIH NIAID SAVE Early Detection Group regularly prioritizes SARS-CoV-2 variants. The main goal of prioritization is to identify relevant lineages for phenotypic testing by experimental groups within the NIH SAVE network by integrating up-to-date epidemiologic, structural, and genetic information. Practically, this process involves regular contact with the experimental groups in weekly meetings and continued consideration of the types of experimental work the groups conduct, which includes characterization for replicative fitness, pathogenicity, and antigenic escape.

The SAVE Early Detection Group does not attempt to recapitulate the efforts of numerous public health organizations worldwide to designate variants of concern and variants of interest (*1*–*3*). The group aims to horizon scan for variants and substitutions; it focuses on early signals of lineages and substitutions that pose a public health threat on the basis of epidemiology and estimated phenotype and prepares for emergence of such lineages by providing information about relevant aspects of SARS-CoV-2 biology to enable faster assessment of future lineages.

The group publishes monthly lineage prioritizations for use by experimental collaborators. Identifying and prioritizing relevant lineages is difficult, and the group draws on the expertise of 9 constituent subgroups. The subgroups take different approaches to prioritizing variants, the results from which are pooled to form publicly available consensus rankings. Since April 2021, the group has produced 21 prioritizations.

## Methods

### Generating Consensus Rankings

Participating groups suggest new lineages, which may be novel lineages or sublineages containing specific additional substitutions on top of a designated Pango (*4*) lineage. For each lineage, the suggesting group provides information about lineage name, substitutions, GISAID/GenBank accession numbers, number of sequences, epidemiologic information, and reason (Table 1).

**Table 1:** Generating consensus rankings for US National Institutes of Health prioritization of SARS-CoV-2 variants

Lineage data	Description
Lineage name	A Pango lineage designation, potentially with specific additional mutations (e.g., BA.5 + R346T).
Substitutions	Substitutions in spike relative to the Wuhan-1 reference genome. Lineages are defined by using the spike sequence because spike mutations are likely to be most relevant for the phenotypic properties the consortium is interested in, such as antibody escape.
GISAID/GenBank accession no.	Example GISAID or GenBank ID that matches the nominated lineage. This provides an example of an intact gene for experimental groups, and acts as a bioinformatic check that the sequence exists.
No. sequences	The number of sequences in GISAID (*5*) matching the lineage.
Epidemiologic information	The number of sequences in each of the countries in which the lineage is most frequently detected.
Reason	Additional information, including structural insights or further epidemiologic information.

### Ranking of Variants

Each group ranks newly suggested lineages and lineages from the previous prioritization (to incorporate new epidemiologic information). Four types of rank can be assigned (Table 2).

**Table 2:** Ranking of variants for US National Institutes of Health prioritization of SARS-CoV-2 variants

Rank	Description
Numeric rank	Ties are permitted
Defer	If a group feels unable to rank a particular lineage
Low	If a group considers a lineage unimportant and does not want to assign a specific numeric rank
Lowest	If a group considers a lineage highly unimportant and does not want to assign a specific numeric rank

### Generation of Consensus Ranking

Ranks are first transformed to numeric ranks (Table 3). Lineages are then ordered by mean rank to produce a consensus, which is split into prioritization categories. Generally, category 1 includes lineages that are either increasing or at high frequency or contain substitutions with evidence of a relevant phenotypic effect. Category 2 contains lineages that are potentially interesting because of epidemiology or sequence, and categories 3 and 4 (when present) are typically a record of other circulating minor lineages. Furthermore, lineages being studied by experimental groups are moved to the ongoing category, and lineages with substantial phenotypic data already available are moved into the well-studied category. After discussion within the Early Detection Group, the consensus ranking is made publicly available (*6*).

**Table 3:** Generation of consensus ranking for US National Institutes of Health prioritization of SARS-CoV-2 variants

Rank	Description
Numeric	The value of the rank. For ties, the mean of the tied range (if the fourth, fifth, and sixth lineages are tied, a rank of 5 is assigned to each.)
Defer	Excluded from average rank
Low	Counted as tied and placed below lineages with numeric or deferred ranks
Lowest	Counted as tied and placed below lineages with numeric, low, or deferred ranks:

### Emergency Updates

Lineages that come to the group’s attention in between prioritizations can be added outside of the normal monthly timeline. They are added to a rank determined directly by discussion between the subgroups.

### Individual Ranking Methods

Ranking methods are presented in general terms because ranking is often performed by hand rather than algorithmically. Manual ranking is performed because of the need to weigh numerous factors, including which types of variation are most interesting to characterize at any particular time.

**The 9 Teams**

The Los Alamos National Laboratory (LANL) team, led by author B.K., developed tools for early detection of spike variants (https://cov.lanl.gov) (*7*), based on emergent mutational patterns in sequences regularly updated from GISAID (https://www.gisaid.org). Because spike protein variants are often found in multiple Pango lineages (sometimes because of recombination [*8*]), and because Pango lineages sometimes include very diverse forms of spike protein, variant dynamics and regional frequency calculations are based on spike sequence rather than Pango lineage. Variant dynamics and global spread are tracked at multiple geographic levels, and variants are deemed to be of interest if relative sampling frequency is substantially increasing in multiple locations or if they are more highly mutated relative to past variants and increasing in >1 geographic location. The relative importance of mutational patterns is weighed by substantial transitions in variant frequencies over time at different geographic levels, structural considerations, levels of convergence, and literature-based assessments of relevance to neutralizing antibody sensitivity and infectivity. The LANL team maintains a folder in the download section on GISAID that contains the information used for suggesting new lineages and provides full-length genome and spike alignments for representative forms of circulating variants.

The University of California Riverside School of Medicine team, led by author A.G., uses relative growth in the prevalence of specific substitutions and deletions/insertions, which are mapped onto Pango lineages or used to define new ones, identifying the fastest growing variants and mutation combinations within these lineages. Although the main focus has been on spike mutations, nonspike mutations are also tracked. These criteria are automated and available from a regularly updated website (https://coronavirus3d.org). For the final variant and subvariant ranking, additional criteria are included: simultaneous growth in >2 distinct geographic locations, mutations in different regions of the spike (N terminal domain [NTD], receptor-binding domain [RBD], furin cleavage region, S2 spike domain), their potential effect on protein structure (by modeling), and the reemergence of individual mutations in novel combinations.

The Bacterial and Viral Bioinformatics Resource Center team, led by author R.S. at the J. Craig Venter Institute, uses a custom heuristic algorithm that combines sequence prevalence metrics with functional impact predictions, focusing on sequence features of concern with the spike protein. To identify concerning upward trends and their global spread for each residue, variant and lineage sequence prevalence and fold growth are calculated month to month in all countries. Substitutions are given a functional impact score based on whether the substitution has been demonstrated to cause a substantial decrease in polyclonal or monoclonal antibody binding, an increase in angiotensin-converting enzyme 2 (ACE2) binding, or if the position is located within the NTD supersite or the furin cleavage site (*9*). The sequence prevalence and functional impact scores are combined to generate an Emergence Score for the ranking of emerging lineages. Although the main focus has been on spike mutations, nonspike mutations are also tracked. A detailed description of the method has been published (*10*).

The Cambridge University team, led by author S.T., follows sequence prevalence increases over time and geographic spread as well as prevalence increases in defined pre-immunized cohorts. This team prioritizes mutations displaying convergent evolution, focusing on those likely to cause immune escape, by looking at experimentally determined antibody escape (with particular focus on polyclonal serum) and by considering structural reasoning. This information is jointly considered when determining the importance of each mutation. The emphasis is primarily on substitutions in RBD and the mechanism around ACE2 binding and secondarily on NTD and proximity to the furin cleavage site. These substitutions are given higher priority if they are clearly transmitting faster and if they are in a different Barnes class from substitutions previously seen in the same lineages but are given lower priority if they have already been characterized.

The Broad Institute team, led by author J.L., believes, like the University of California-Riverside School of Medicine team, that the accelerated growth of a lineage relative to its peers, across multiple geographic regions, is the single most important marker of lineages of potential concern. The team has developed 2 related approaches for identifying such lineages. The first approach fits a binomial logistic regression to the proportion of each lineage over time in every state. The team then systematically compares the growth rate of every lineage in each state, relative to all other lineages, and identifies lineages that are consistently increasing in multiple states (e.g., as in Earnest et al. [*11*]). A second related approach fits multinomial logistic regression models across geographic regions (*12*). This approach is a generalization of the first approach that estimates the relative growth rate of each lineage compared with every other, allowing for more complex and realistic lineage dynamics such as nonmonotonic modeled trajectories. The results of these 2 approaches form the basis for an initial prioritization list that is then discussed internally and brought to NIH SAVE discussions.

The Walter Reed Army Institute of Research team, led by author M.R., has a variant scoring scheme based on increased prevalence and potential effects of mutations in spike. This scoring is primarily performed in a lineage-independent manner; hence, the initial focus is on convergent evolution rather than on lineage tracking, although changing mutation frequencies within lineages are also tracked. Weight scores are given for various characteristics such as fold increase over time, geographic spread, variant growth, and potential effects on antibody recognition. Relevant antibody contact sites in the NTD and RBD are identified by analyzing spike–antibody complex structures deposited in the Protein Data Bank (http://www.wwpdb.org). The identification of contact sites is performed to upweight substitutions at relevant antibody binding sites with recent changes in frequency. Several tools enable tracking of variants of concern and mutations of interest at global, regional, and country levels on a weekly basis by using data from the previous 3 months. An open-build using data from GenBank is available (*13*).

 The Icahn School of Medicine at Mt. Sinai team, led by author H.v.B., has a similar approach to the Walter Reed Army Institute of Research team, ranking variants based on an aggregate score for sequence prevalence increase and genetic changes of concern, but the criteria differ slightly for different genomic regions. A higher weight is given to mutations associated with antibody escape or changes in ACE2 affinity, with a focus on NTD and RBD, associated with higher transmissibility, with evidence of convergent evolution, near the furin cleavage site, and in enzyme active sites. Moreover, data from surveillance cohorts in the New York, New York, metropolitan area are used to assess lineages associated with breakthrough infections after vaccination. To minimize false positives and increase confidence of early detection, historical data are used to estimate weighting factors and to add or remove criteria.

The Ben Gurion University of the Negev, The National Institute for Biotechnology, in the Negev, Israel, team, led by author T.H., takes an approach based solely on the prediction of potential antibody escape caused by mutations. The team analyzes a large set of solved 3-dimensional antibody structures for spike, curated from the Protein Data Bank. Contact positions for each antibody are extracted on the basis of solved structures. To assess the effects of each single point amino acid mutation on escape from antibody responses, the predicted changes in binding energies (ΔΔG) for each antibody are computed for each specific mutation within its contact footprint, by using FoldX (*14*). Using the ΔΔG scores, team members compute an antibody escape score for each mutation. Variants are then scored and ranked on the basis of the predicted cumulative effect of their mutations on antibody evasion.

The University of Missouri team, led by author M.J., believes that because a minority of prolonged infections give rise to variants containing numerous convergent mutations that arise independently in different locations, these convergent variants forecast those likely to arise in future circulating viruses. These lineages were initially discovered through wastewater sequencing, in which highly divergent SARS-CoV-2 lineages (cryptic lineages) were sporadically identified (*15*,*16*). A few similarly advanced lineages have now also been found from long-term COVID-19 patients. The team maintains a database of evolutionarily advanced cryptic lineages and evolutionarily advanced patient lineages and has identified numerous discrete mutations repeatedly appearing in these advanced lineages. Indeed, all prominent amino acid changes in the RBD of the dominant Omicron sublineages were observed repeatedly in evolutionarily advanced lineages before appearing in Omicron. The team systematically evaluates new lineages by comparing combinations of changes in new circulating lineages with those that have appeared frequently in advanced lineages and reports their findings during NIH SAVE discussions.

## Results

**Figure 1:**
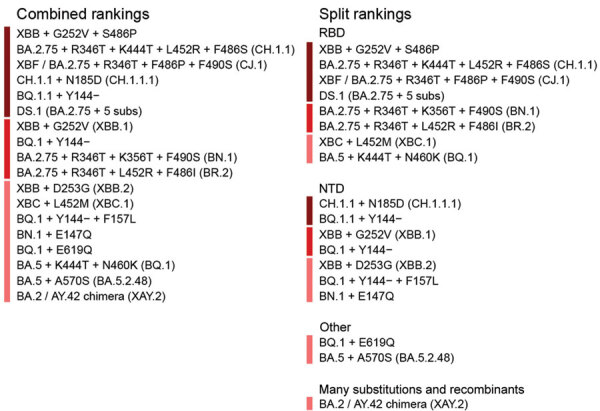
US National Institutes of Health National Institute of Allergy and Infectious Diseases SARS-CoV-2 Assessment of Viral Evolution Early Detection consortium prioritization of variant rankings from February 2023, produced by taking the consensus of rankings provided by the consortium subgroups. The lineages are shown on the right, split into different functional categories. The colored bars indicate priority categories 1, 2, and 3. This prioritization, and all future prioritizations, can be accessed with supporting information (*6*) (Appendix 1).

We display the February 2023 prioritization in priority order and split into functional categories (*6*) (Figure 1; Appendix 1). Functional groups are based on the region of spike harboring mutations in each lineage (RBD, NTD, or other). Lineages are placed in the many substitutions or recombinants section if they contain many substitutions relative to other circulating variants or were produced by recombination. The split enables experimental groups to quickly identify and compare the lineages most relevant to their focus. The split also alleviates a difficulty of using the consensus-approach to prioritization. Different groups use different methods to rank lineages; some focus primarily on epidemiologic data; others focus on whether a lineage contains mutations that are likely to have particular phenotypic effects or that show substantial convergent evolution. Furthermore, groups focus on different regions of the spike protein; because mutations in different regions are likely to have different phenotypic effects, prioritizing between them is difficult. Splitting the prioritization into structural regions makes it easier to compare lineages that have been nominated for similar reasons.

**Figure 2:**
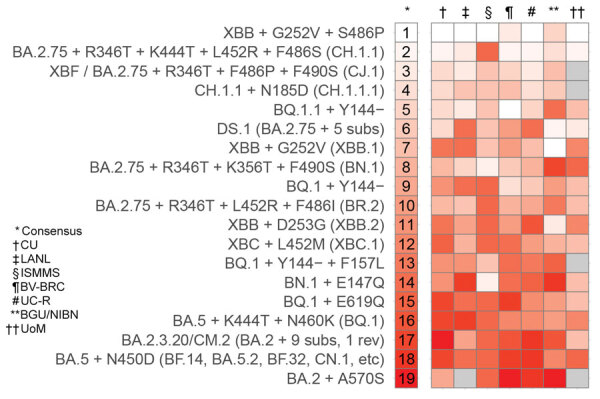
Comparison of rankings provided by 7 of the subgroups of the US National Institutes of Health National Institute of Allergy and Infectious Diseases SARS-CoV-2 Assessment of Viral Evolution consortium for the February 2023 prioritization. Lighter colors indicate a higher ranking and darker colors a lower ranking; gray indicates that the group deferred or did not provide a ranking for that lineage. Two groups (Broad Institute team, Walter Reed Army Institute of Research team) were not able to provide rankings for the February 2023 prioritization. Appendix 3 Figure 1 shows tangle plots comparing rankings between all pairs of consortium subgroups. BGU/NIBN, Ben Gurion University of the Negev, The National Institute for Biotechnology in the Negev; BV-BRC, Bacterial and Viral Bioinformatics Resource Center; CU, Cambridge University; LANL, Los Alamos National Laboratory; ISMMS, Icahn School of Medicine at Mt. Sinai; UC-R, University of California-Riverside School of Medicine; UoM, University of Missouri.

We also compared the rankings provided by each subgroup for the February 2023 prioritization (Figure 2). We found generally good agreement between subgroups, although with some notable differences. For example, the DS.1 lineage is ranked higher by the Ben Gurion University of the Negev team than by other subgroups. This finding is consistent with their ranking method, which focuses on antibody escape, given that DS.1 contains 5 RBD substitutions on top of the BA.2.75 spike sequence. By contrast, it is ranked lower by groups focusing on epidemiology, because of its low observed count.

We also compared the 4 most recent prioritizations, showing the movement of lineages between priority categories (Appendix 2). Category 1 lineages, which typically show consistent growth or contain phenotypically relevant substitutions, are retained in the following month’s prioritization 98% of the time, compared with 67% for category 2, and 38% for category 3, and 28% for category 4 (Appendix 3 Figure 2). Indeed, on only 2 occasions have lineages in categories 3 or 4 later entered the ongoing or well-studied categories, when AY.1 and AY.2 did so during July–November 2021. Lineages rarely move from a lower to a higher prioritization category (Appendix 3 Figure 2), suggesting that high priority lineages are typically judged to need immediate characterization. Similarly, lineages that the group has been aware of without considering them highly important are unlikely to become substantially more important over time, suggesting that newer lineages should perhaps be considered for characterization.

**Figure 3:**
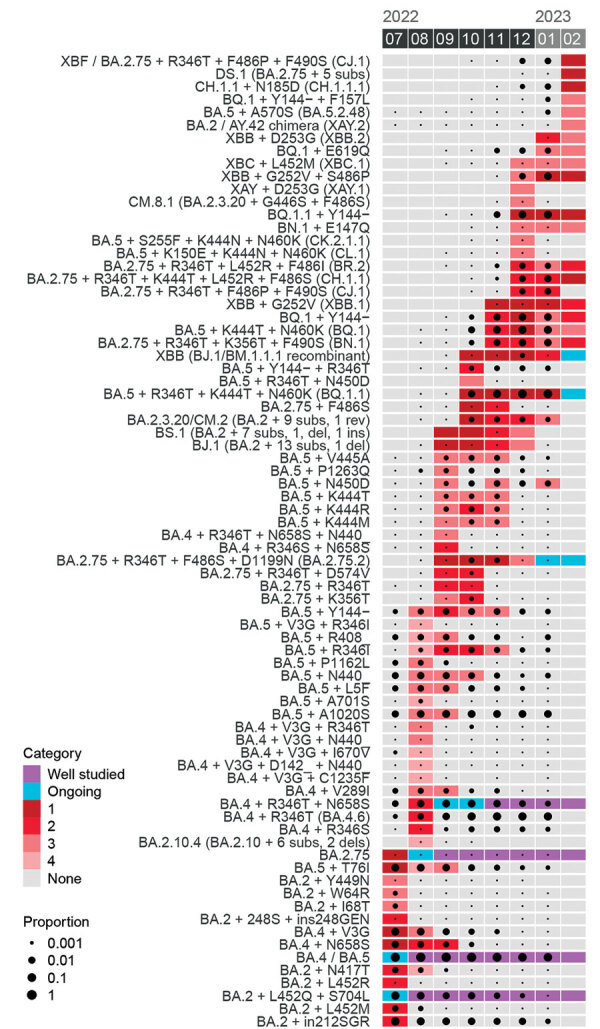
Gantt chart showing the priority category (denoted by colored rectangles) for each SARS-CoV-2 lineage at each monthly prioritization between May 2022 and February 2023. The frequency of each lineage in global surveillance data is shown with black circles for each month. For lineages with a frequency <0.1%, but which are observed at least once in a given month, a small black point is shown. Exact matches to sequence definitions as provided in US National Institutes of Health National Institute of Allergy and Infectious Diseases SARS-CoV-2 Assessment of Viral Evolution Early Detection consortium prioritization spreadsheets are required for a sequence to count as a match to a lineage. Appendix 3 Figure 3 is an analogous figure for all 21 monthly prioritizations, back to April 2021.

Comparing the prioritization categories to epidemiologic data (Figure 3; Appendix 3 Figure 3) shows that lineages are generally identified before or shortly after they reach 0.1% of global circulation. However, the prioritizations do not attempt to predict or recapitulate the epidemiology of lineages but attempt rather to identify relevant lineages to study, weighing the probability that a variant circulates widely, the risk it would pose should it do so, and our ability to estimate its phenotype based on existing data. Lineages can therefore circulate at moderately high frequency without being highly prioritized because their constituent substitutions are less likely to have relevant phenotypic effects (e.g., BA.5 + A1020S) or because the lineage’s high frequency seems to be caused by founder effects.

## Discussion

Producing prioritizations has been successful in 2 respects. First, and of primary concern, the prioritizations have provided experimental groups within NIH SAVE easy access to regularly updated information on the recent evolution and epidemiology of SARS-CoV-2, used to guide phenotypic investigations. In addition to simply giving access to raw data, which are now accessible by using numerous online epidemiologic tools, the prioritizations are synthesized with close consideration of the interests of the experimental groups and consider numerous factors in assessing the relevance of circulating lineages.

Second, most major variants of SARS-CoV-2 have been rapidly identified by the SAVE Early Detection Group and include Delta and Lambda (in the first prioritization in April 2021), Mu (in July 2021), and Omicron as an emergency update (between the November and December 2021 prioritizations). Indeed, the usefulness of the rankings has increased over time; Omicron lineages BA.2 and BA.5 have diversified into a wide range of sublineages, which have been epidemiologically successful (showing growth in numerous locations) and often show considerable immune escape, making them key lineages to track for vaccine effectiveness purposes (*17*). Those lineages have been rapidly identified and tracked in the prioritizations, including BF.7 (June 2022), BA.2.75 (July 2022), BQ.1.1 (October 2022), XBB (October 2022), and BN.1 (November 2022).

The prioritizations have been most useful to experimental collaborators in this period of high diversity, helping to identify adaptive and threatening variation, which is most pressing for study. By contrast, variation for identifying and prioritizing in earlier periods of the pandemic, such as during the circulation of Delta, was less epidemiologically useful. This period of rapid diversification has also made clear the value of the consensus approach; with each subgroup routinely monitoring the evolution of the virus from its own perspective, a well-rounded assessment of new variants can be rapidly reached.

This hands-on approach does come with limitations. It is labor intensive, requiring continued active participation from each research group; it is not an automated system because we do not believe that there is an automated solution for monitoring the ever-changing challenges presented by evolution of SARS-CoV-2. This manual approach also means that the prioritization typically only works at a monthly resolution, although lineages are sometimes added to the prioritization in between monthly updates if deemed necessary.

Of note, 3 major lineage replacements (Alpha, Delta, Omicron) have emerged while another lineage has dominated global circulation. During the period of Alpha and Delta circulation, focus was placed on subvariants of those lineages containing a small number of additional substitutions. Although this focus was in part to enable experimental work to enhance scientific understanding of SARS-CoV-2 phenotypic variation, we suspected that the next dominant strain might have been produced by refinement of the current dominant strain, through addition of further fitness-enhancing substitutions. Our suspicion was based on 2 reasons: first, fitness-enhancing substitutions are likely to appear first in the dominant strain because of its greater circulation; and second, the rapid expansion of Alpha and then Delta demonstrated fitness advantages over the previously circulating strain. Substitutions arising in other strains would need to first overcome this fitness deficit and would be outcompeted by a sublineage of the dominant strain containing the same substitution, disregarding potential epistatic effects.

In reality, emergence of Delta and Omicron has shown that the next dominant strain is not always produced by incremental refinement of the current dominant strain. Whether SARS-CoV-2 will continue to evolve in this fashion is unclear, particularly given the success of numerous more incremental subvariants of Omicron BA.2 and BA.5, which more closely follow the pattern we were preparing for during circulation of Alpha and Delta. Regardless of which of those patterns governs SARS-CoV-2 evolution in the long term, continued surveillance is needed to identify variation within the currently dominant lineage and to detect highly divergent sequences, which may be particularly likely to originate from countries in which a smaller proportion of cases are sequenced, which may harbor substantial unsampled variation; in Delta and Omicron, the emergent lineage was first identified in a country that performed minimal sequencing (Omicron in Botswana) or minimal sequencing relative to circulation (Delta in India).

The prioritizations made by the 9 laboratories that form the NIH Early Detection team have been a valuable resource for helping experimental groups keep up to date with the rapid evolution of SARS-CoV-2. The NIH Early Detection team will continue to refine the methods used by individual subgroups to prioritize variants and the way in which this information is presented to experimental collaborators.

Appendix 1 US National Institutes of Health SARS-CoV-2 assessment of Viral Evolution Early Detection prioritization summary.

Appendix 2 US National Institutes of Health National Institute of Allergy and Infectious Diseases SARS-CoV-2 assessment of Viral Evolution Early Detection consortium prioritization of variant rankings for November 2022–February 2023.

Appendix 3 Additional information for US National Institutes of Health prioritization of SARS-CoV-2 variants.
