# Supplementary material for: US National Institutes of Health Prioritization of SARS-CoV-2 Variants
Source: Emerg Infect Dis. 2023 May;29(5):e221646. doi: 10.3201/eid2905.221646 (PMC10124642; doi:10.3201/eid2905.221646)
Supplement: Appendix 2 — US National Institutes of Health National Institute of Allergy and Infectious Diseases SARS-CoV-2 assessment of Viral Evolution Early Detection consortium prioritization of variant rankings for November 2022–February 2023. [file 22-1646-Techapp-s2.pdf]

Article DOI: https://doi.org/10.3201/eid2905.221646

EID cannot ensure accessibility for supplementary materials supplied by authors. Readers who have difficulty accessing supplementary content should contact the authors for assistance.

US National Institutes of Health Prioritization of SARS-CoV-2 Variants

Appendix 2

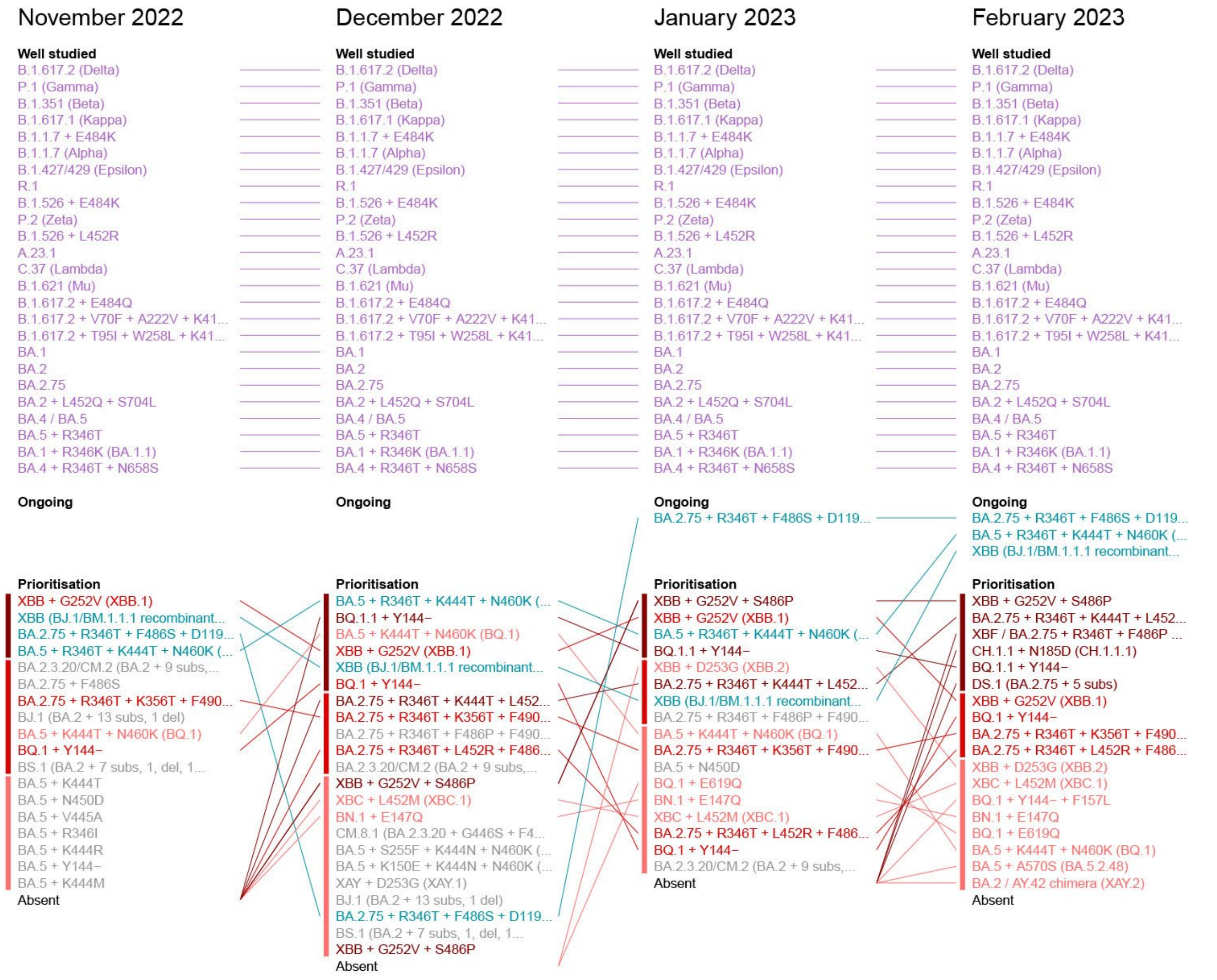

Appendix 2 Figure. US National Institutes of Health National Institute of Allergy and Infectious Diseases SARS-CoV-2 assessment of Viral Evolution Early Detection consortium prioritization of variant rankings for November 2022–February 2023. The prioritizations are produced by taking the consensus of rankings provided by consortium subgroups. Lineages are colored by their prioritization category in the February 2023 ranking (including “well studied” and “ongoing” categories). Equivalent figures colored for each other

month are available in Appendix 3 (https://wwwnc.cdc.gov/EID/article/29/5/22-1646-App3.pdf). Colored bars indicate priority categories 1, 2, and 3 in each month. These prioritizations, and all future prioritizations, can be accessed with supporting information online as outlined in in the main text or in Appendix 1 (https://wwwnc.cdc.gov/EID/article/29/5/22-1646-App1.pdf).
