# Supplementary material for: US National Institutes of Health Prioritization of SARS-CoV-2 Variants
Source: Emerg Infect Dis. 2023 May;29(5):e221646. doi: 10.3201/eid2905.221646 (PMC10124642; doi:10.3201/eid2905.221646)
Supplement: Appendix 3 — Additional information for US National Institutes of Health prioritization of SARS-CoV-2 variants. [file 22-1646-Techapp-s3.pdf]

Article DOI: https://doi.org/10.3201/eid2905.221646

US National Institutes of Health Prioritization of SARS-CoV-2 Variants

Appendix 3

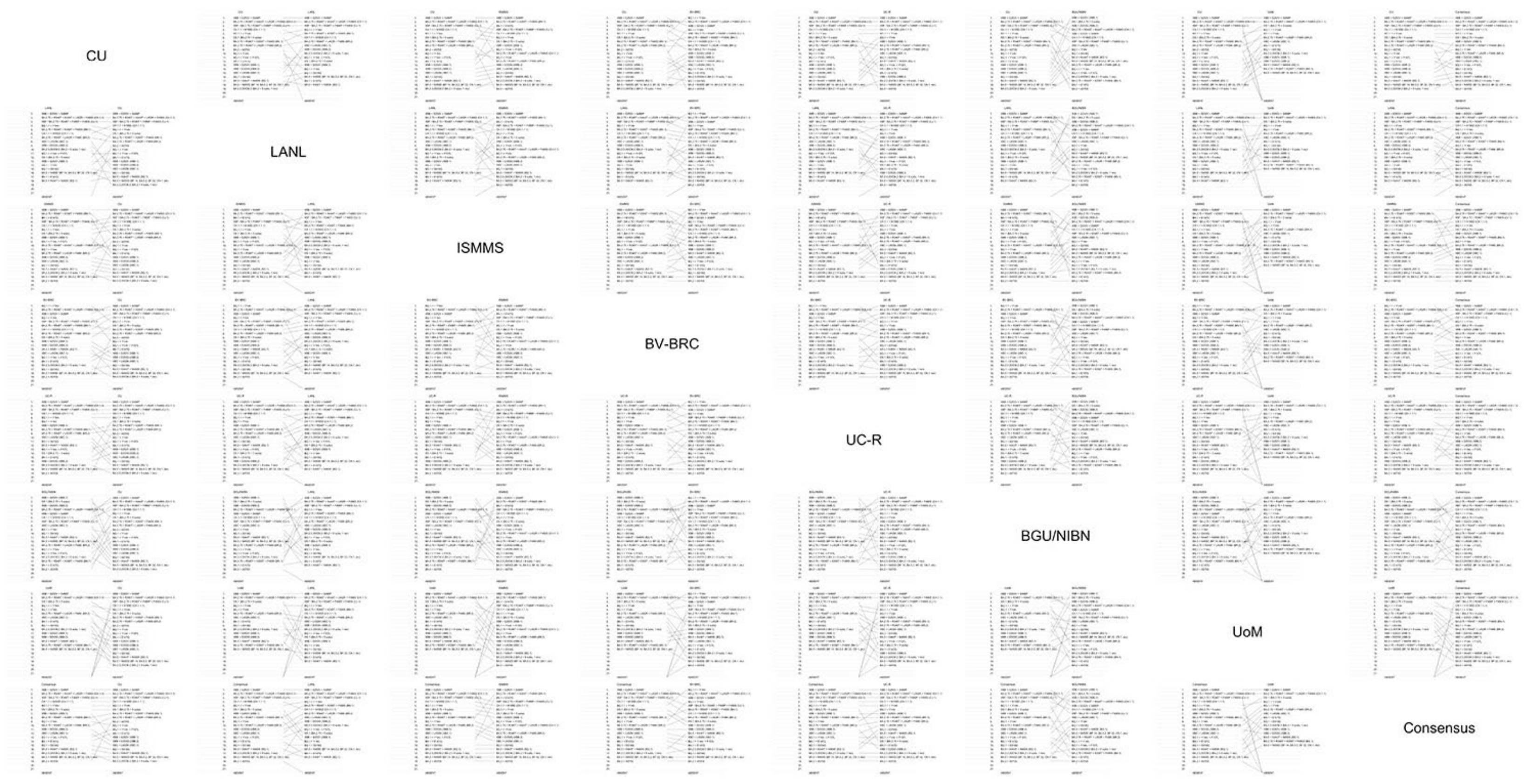

Appendix 3 Figure 1. Pairwise comparison of rankings provided by each of the NIH SAVE Early Detection subgroups, and the consensus ranking, for the February 2023 prioritization. Each subpanel in the figure compares the two sets of rankings. All comparisons against a particular set of rankings can be seen by following the corresponding row or column which intersects with the label for that subgroup.

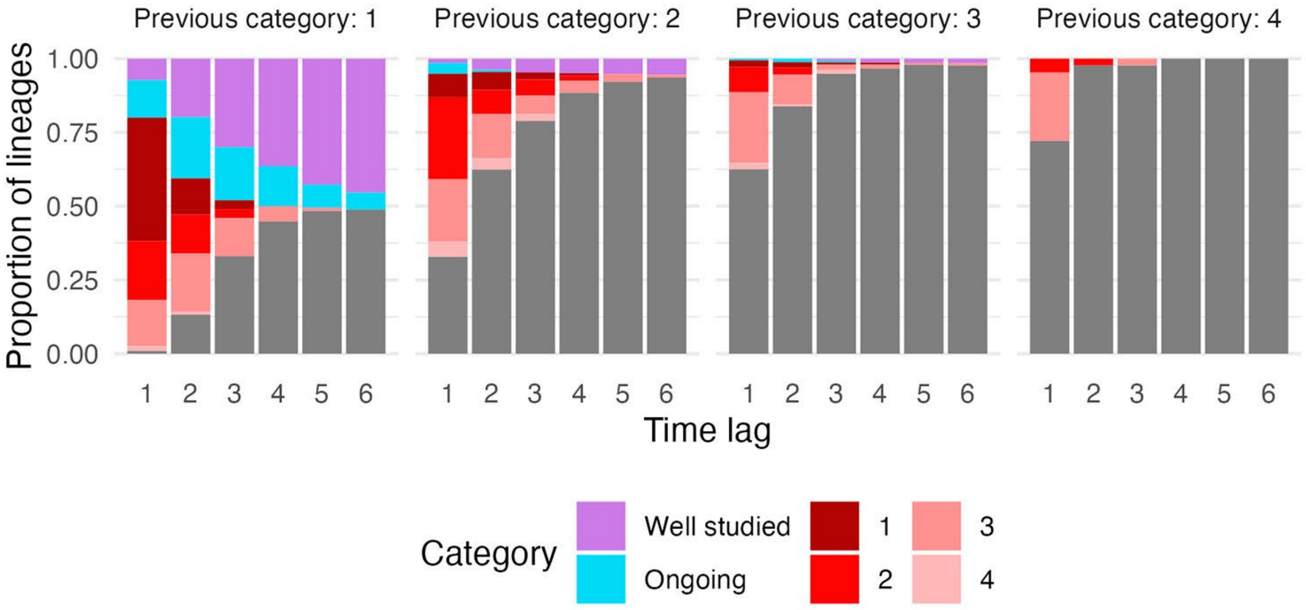

Appendix 3 Figure 2. Figure shows the movement of lineages between prioritization categories over time. For the leftmost set of bars, the first bar shows the distribution of categories for lineages that were in Category 1 in the immediately preceding prioritization. The next bar shows this distribution after 2 months, and so on for subsequent bars. The subsequent three sets of bars shows the same information for lineages that were previously in Categories 2, 3, and 4.

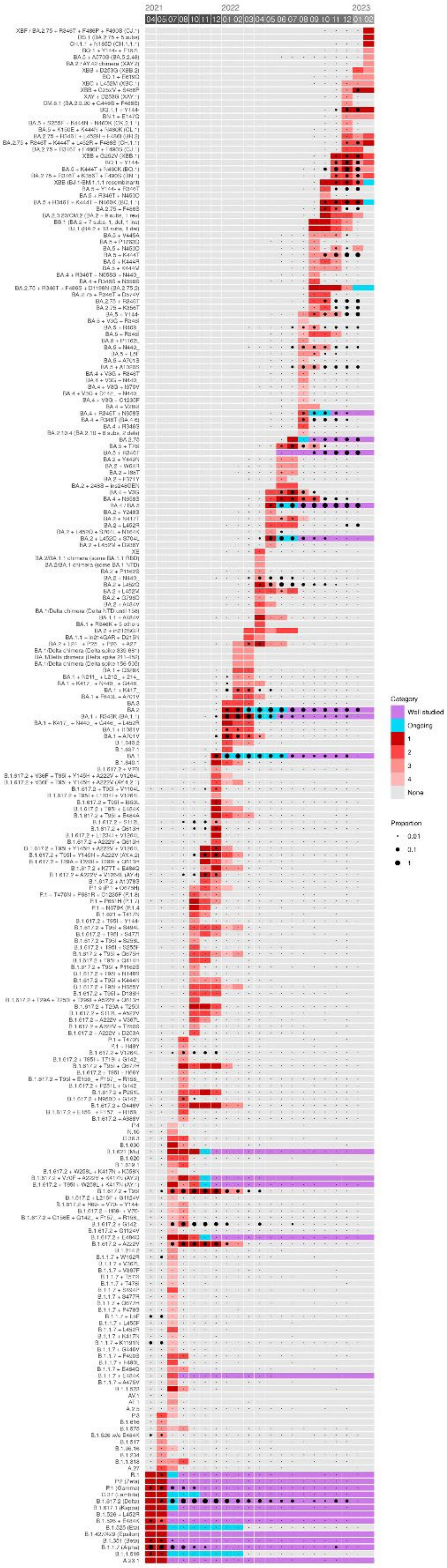

Appendix 3 Figure 3. Equivalent to Figure 4, for all months back to April 2021. Gantt chart showing the priority category (denoted by colored rectangles) achieved by each lineage at in each monthly prioritization between April 2021 and February 2023. The frequency of each lineage in global surveillance data is shown with black circles in each month. For lineages with a frequency of below 0.1%, but which are observed at least once in a given month, a small black point is shown in the diagonal panels.

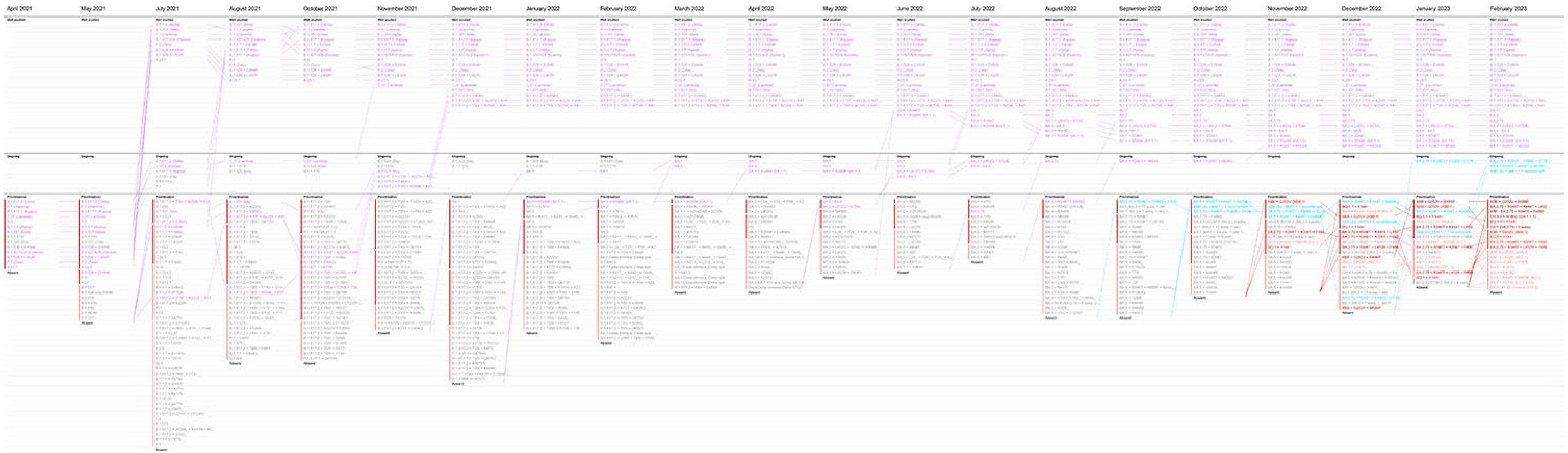

Appendix 3 Figure 4. Analogous to Appendix 2 Figure, for February 2023 prioritization, and showing all prioritizations. NIH SAVE Early Detection Prioritization of Variant rankings from 21 prioritizations between February 2023 and April 2021. The prioritizations are produced by taking the consensus of rankings provided by 9 subgroups which form the NIH SAVE Early Detection Group. Lineages are colored by their prioritization category in the February 2023 ranking. The colored bars indicate priority categories 1, 2, 3, and 4 in each month.

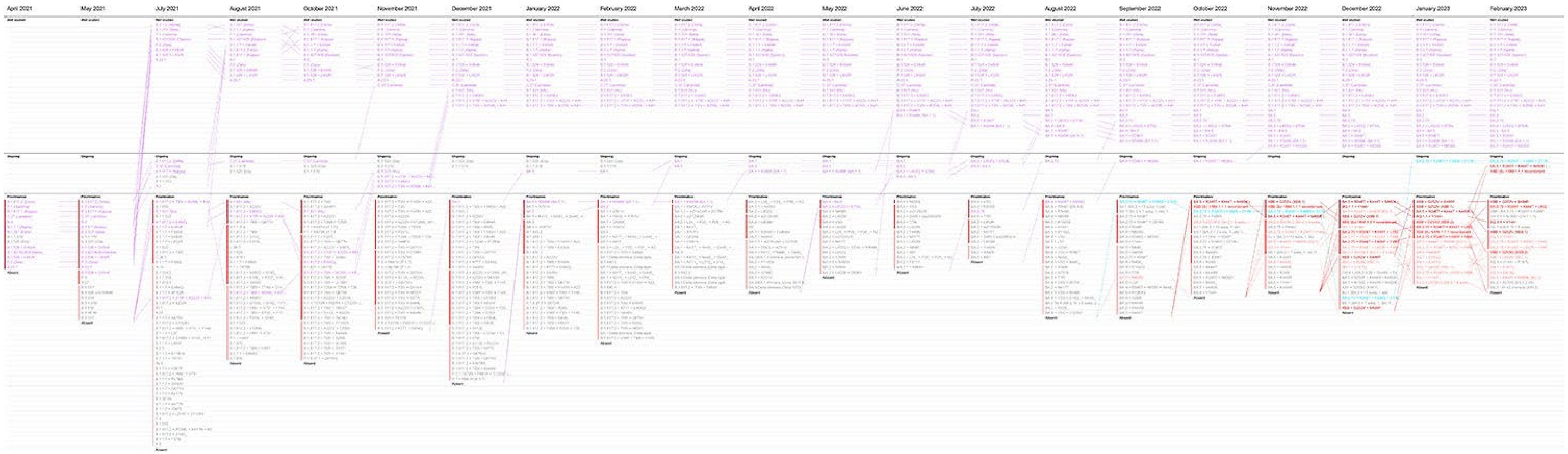

Appendix 3 Figure 5. Analogous to Appendix 2 Figure, for January 2023 prioritization, and showing all prioritizations. NIH SAVE Early Detection Prioritization of Variant rankings from 21 prioritizations between February 2023 and April 2021. The prioritizations are produced by taking the consensus of rankings provided by 9 subgroups which form the NIH SAVE Early Detection Group. Lineages are colored by their prioritization category in the January 2023 ranking. The colored bars indicate priority categories 1, 2, 3, and 4 in each month.

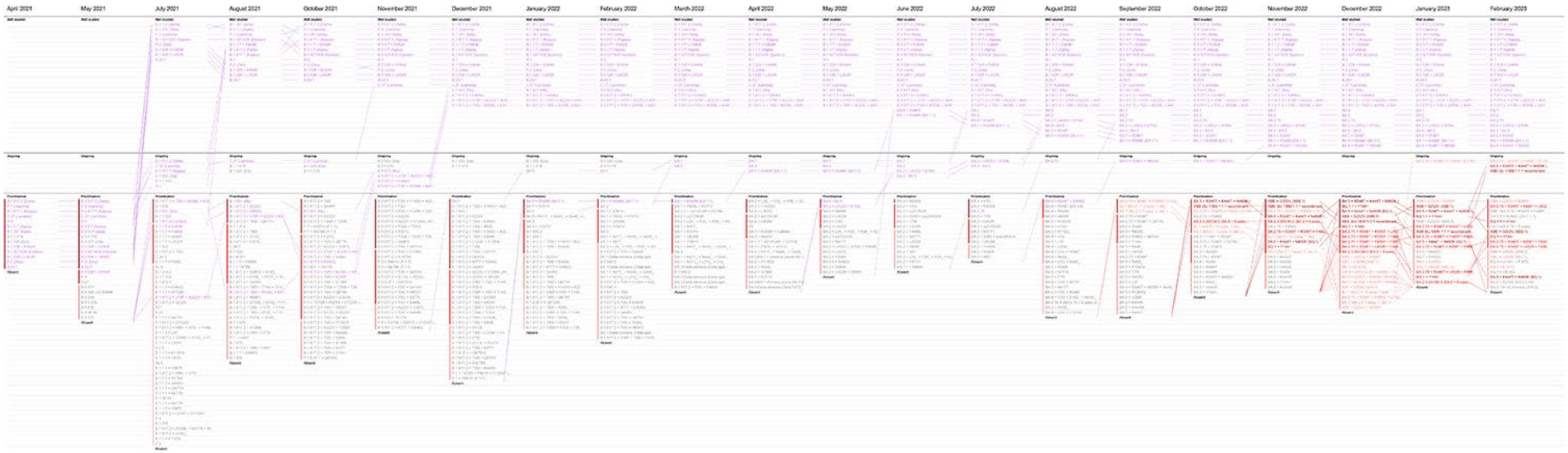

Appendix 3 Figure 6. Analogous to Appendix 2 Figure, for December 2022 prioritization, and showing all prioritizations. NIH SAVE Early Detection Prioritization of Variant rankings from 21 prioritizations between February 2023 and April 2021. The prioritizations are produced by taking the consensus of rankings provided by 9 subgroups which form the NIH SAVE Early Detection Group. Lineages are colored by their prioritization category in the December 2022 ranking. The colored bars indicate priority categories 1, 2, 3, and 4 in each month.

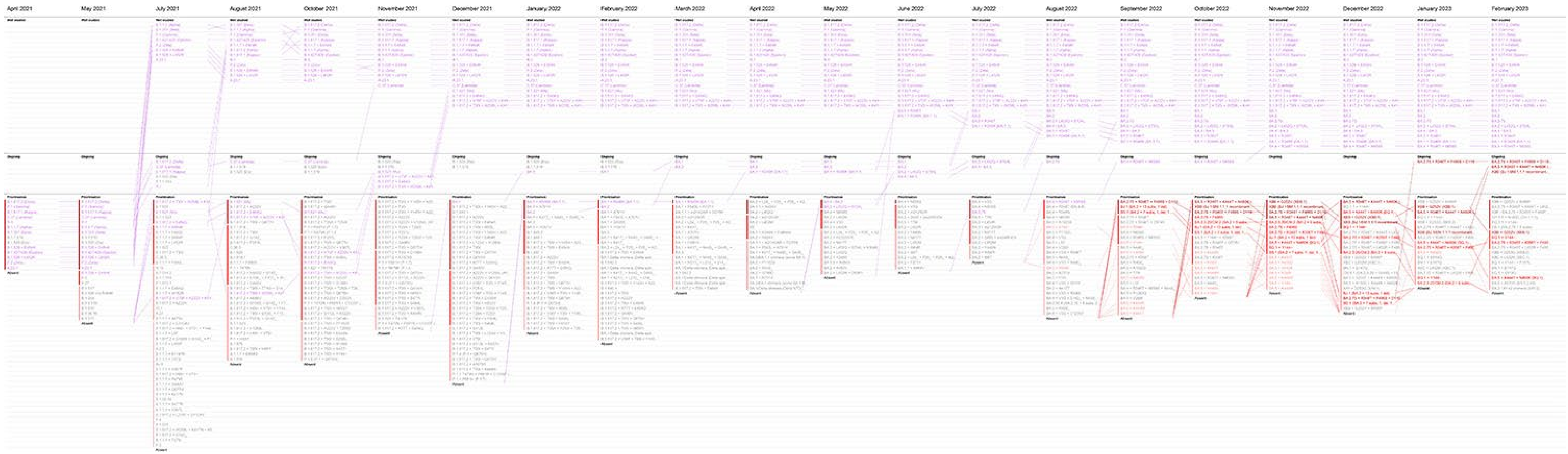

Appendix 3 Figure 7. Analogous to Appendix 2 Figure, for November 2022 prioritization, and showing all prioritizations. NIH SAVE Early Detection Prioritization of Variant rankings from 21 prioritizations between February 2023 and April 2021. The prioritizations are produced by taking the consensus of rankings provided by 9 subgroups which form the NIH SAVE Early Detection Group. Lineages are colored by their prioritization category in the November 2022 ranking. The colored bars indicate priority categories 1, 2, 3, and 4 in each month.

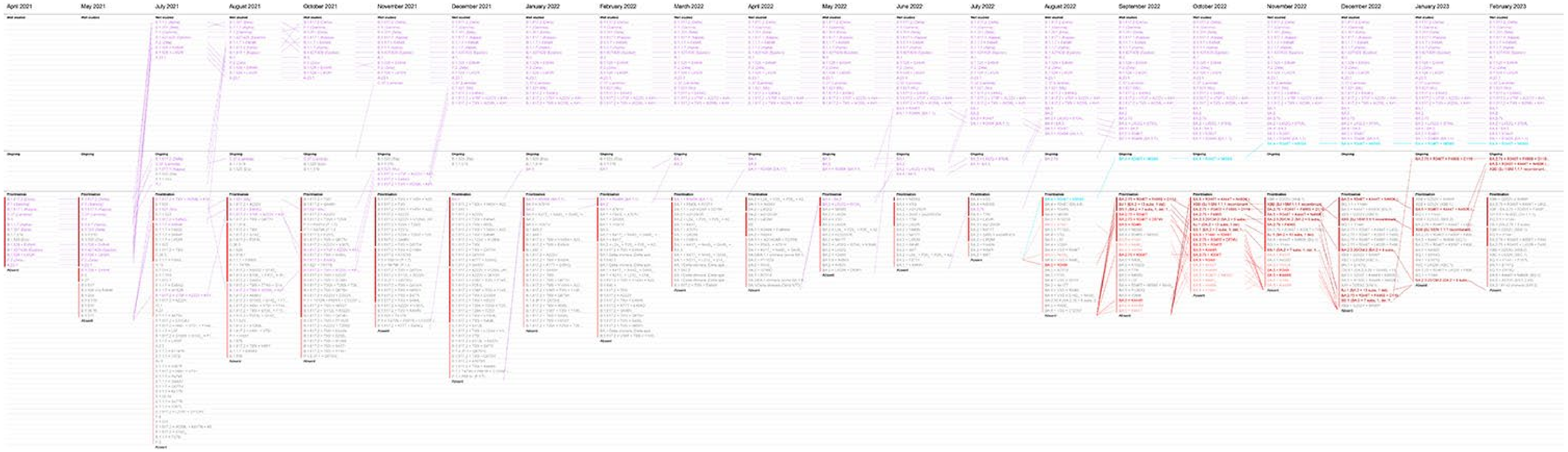

Appendix 3 Figure 8. Analogous to Appendix 2 Figure, for October 2022 prioritization, and showing all prioritizations. NIH SAVE Early Detection Prioritization of Variant rankings from 21 prioritizations between February 2023 and April 2021. The prioritizations are produced by taking the consensus of rankings provided by 9 subgroups which form the NIH SAVE Early Detection Group. Lineages are colored by their prioritization category in the October 2022 ranking. The colored bars indicate priority categories 1, 2, 3, and 4 in each month.

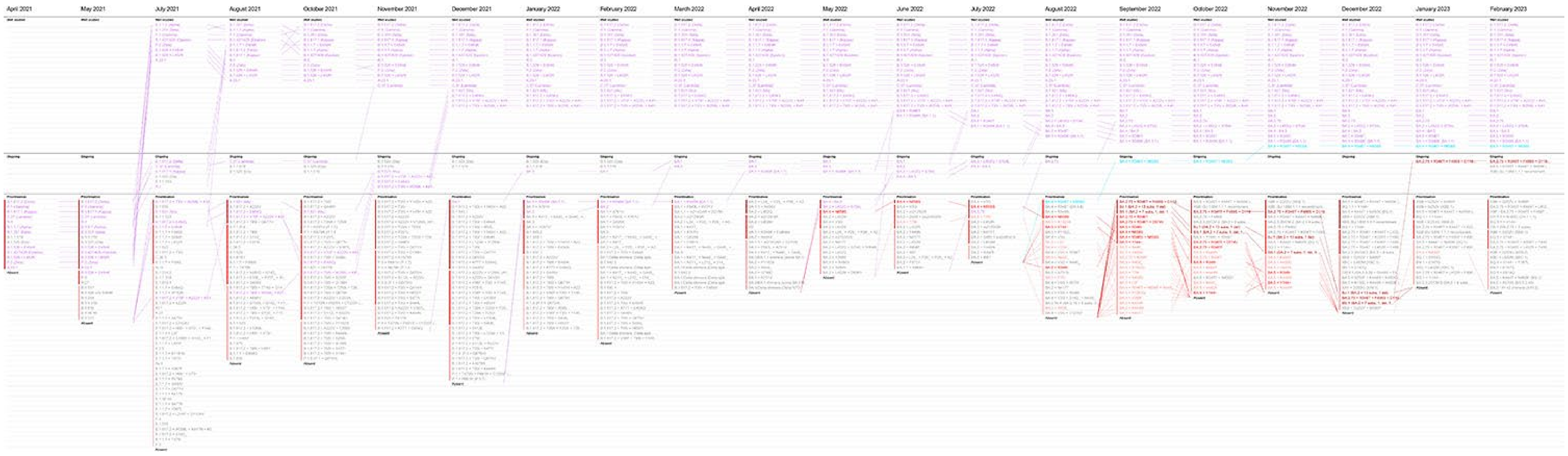

Appendix 3 Figure 9. Analogous to Appendix 2 Figure, for September 2022 prioritization, and showing all prioritizations. NIH SAVE Early Detection Prioritization of Variant rankings from 21 prioritizations between February 2023 and April 2021. The prioritizations are produced by taking the consensus of rankings provided by 9 subgroups which form the NIH SAVE Early Detection Group. Lineages are colored by their prioritization category in the September 2022 ranking. The colored bars indicate priority categories 1, 2, 3, and 4 in each month.

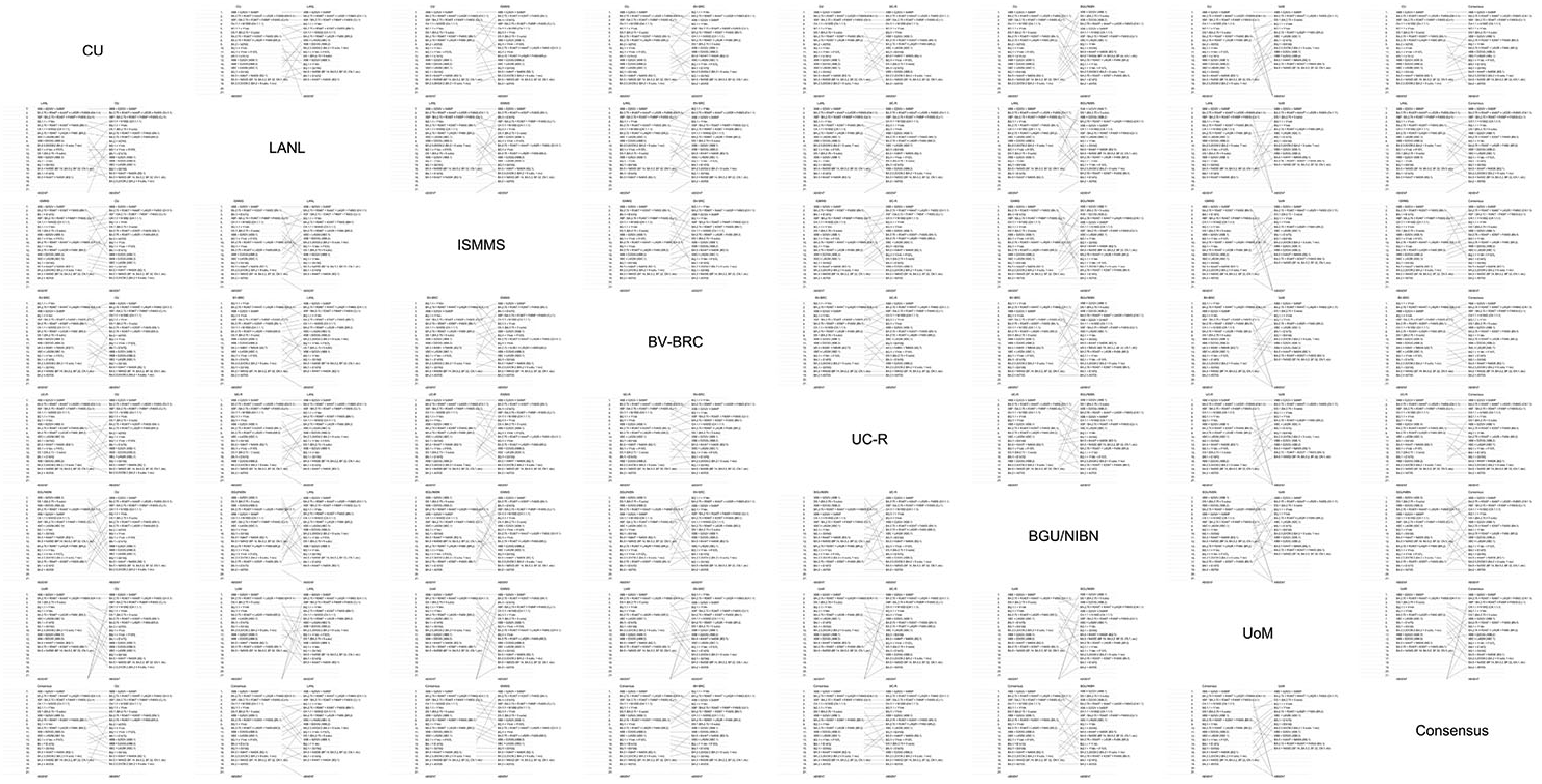

Appendix 3 Figure 10. Analogous to Appendix 2 Figure, for August 2022 prioritization, and showing all prioritizations. NIH SAVE Early Detection Prioritization of Variant rankings from 21 prioritizations between February 2023 and April 2021. The prioritizations are produced by taking the consensus of rankings provided by 9 subgroups which form the NIH SAVE Early Detection Group. Lineages are colored by their prioritization category in the August 2022 ranking. The colored bars indicate priority categories 1, 2, 3, and 4 in each month.

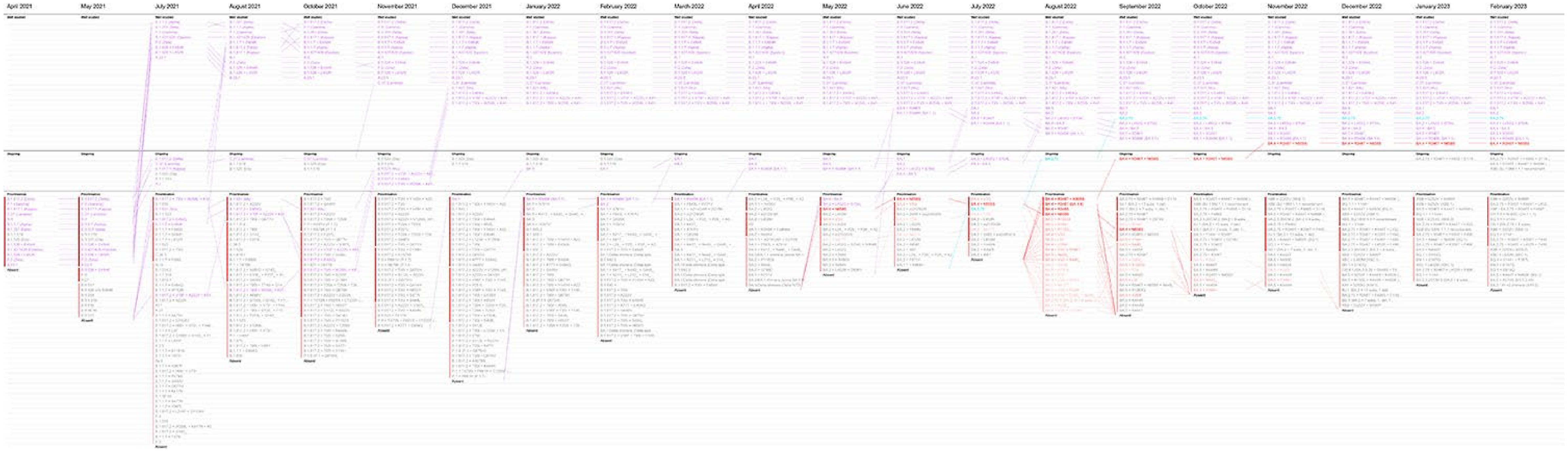

Appendix 3 Figure 11. Analogous to Appendix 2 Figure, for July 2022 prioritization, and showing all prioritizations. NIH SAVE Early Detection Prioritization of Variant rankings from 21 prioritizations between February 2023 and April 2021. The prioritizations are produced by taking the consensus of rankings provided by 9 subgroups which form the NIH SAVE Early Detection Group. Lineages are colored by their prioritization category in the July 2022 ranking. The colored bars indicate priority categories 1, 2, 3, and 4 in each month.

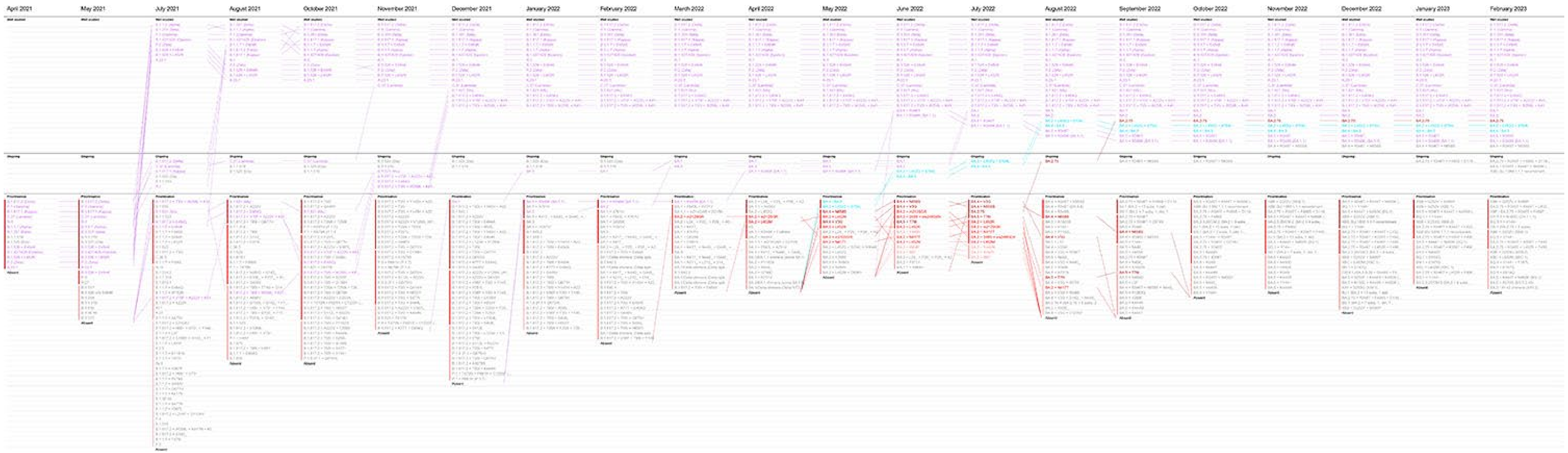

Appendix 3 Figure 12. Analogous to Appendix 2 Figure, for June 2022 prioritization, and showing all prioritizations. NIH SAVE Early Detection Prioritization of Variant rankings from 21 prioritizations between February 2023 and April 2021. The prioritizations are produced by taking the consensus of rankings provided by 9 subgroups which form the NIH SAVE Early Detection Group. Lineages are colored by their prioritization category in the June 2022 ranking. The colored bars indicate priority categories 1, 2, 3, and 4 in each month.

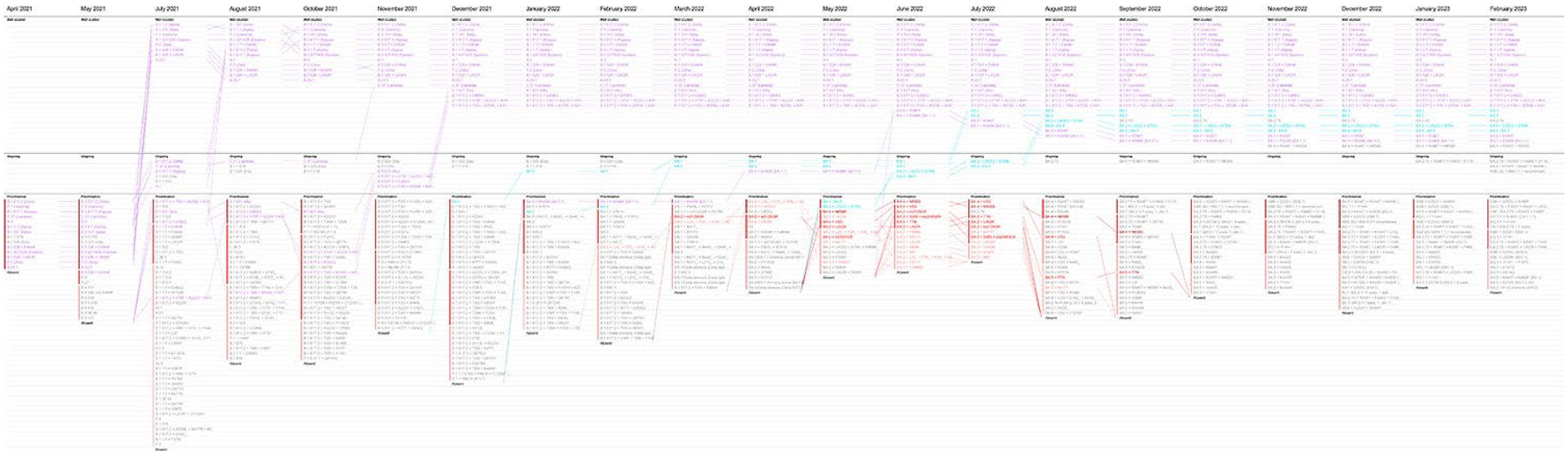

Appendix 3 Figure 13. Analogous to Appendix 2 Figure, for May 2022 prioritization, and showing all prioritizations. NIH SAVE Early Detection Prioritization of Variant rankings from 21 prioritizations between February 2023 and April 2021. The prioritizations are produced by taking the consensus of rankings provided by 9 subgroups which form the NIH SAVE Early Detection Group. Lineages are colored by their prioritization category in the May 2022 ranking. The colored bars indicate priority categories 1, 2, 3, and 4 in each month.

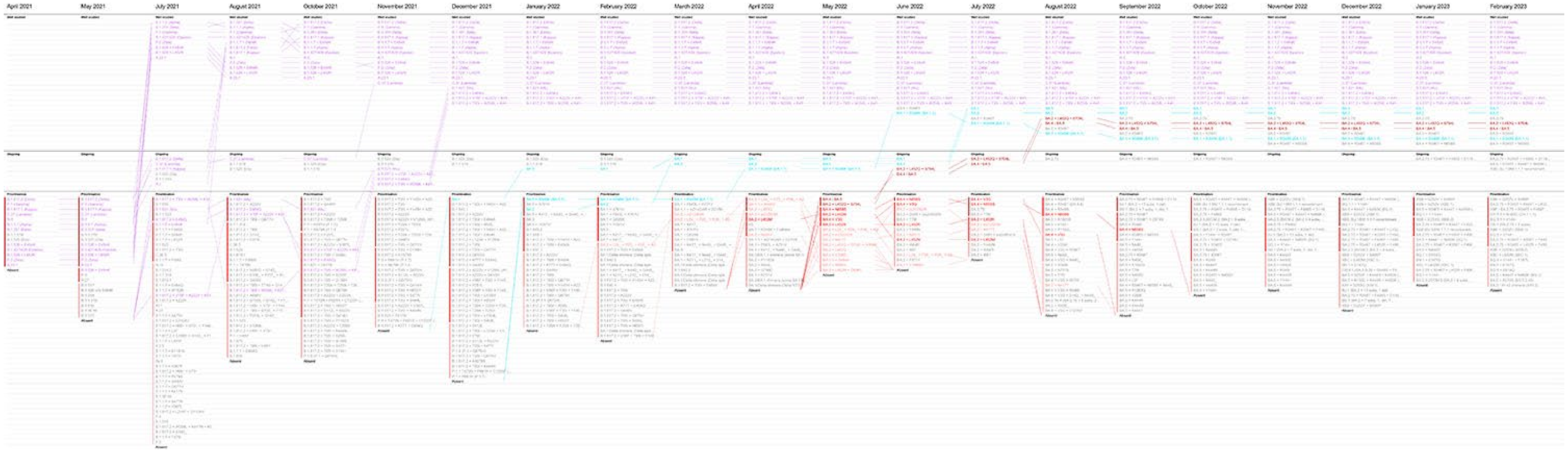

Appendix 3 Figure 14. Analogous to Appendix 2 Figure, for April 2022 prioritization, and showing all prioritizations. NIH SAVE Early Detection Prioritization of Variant rankings from 21 prioritizations between February 2023 and April 2021. The prioritizations are produced by taking the consensus of rankings provided by 9 subgroups which form the NIH SAVE Early Detection Group. Lineages are colored by their prioritization category in the April 2022 ranking. The colored bars indicate priority categories 1, 2, 3, and 4 in each month.

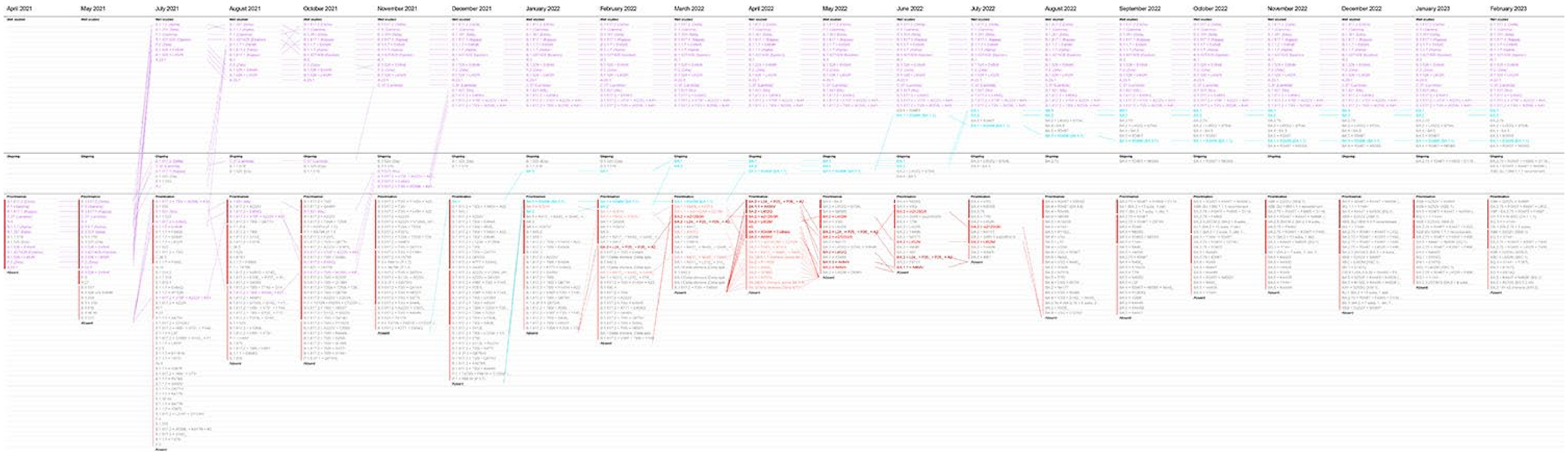

Appendix 3 Figure 15. Analogous to Appendix 2 Figure, for March 2022 prioritization, and showing all prioritizations. NIH SAVE Early Detection Prioritization of Variant rankings from 21 prioritizations between February 2023 and April 2021. The prioritizations are produced by taking the consensus of rankings provided by 9 subgroups which form the NIH SAVE Early Detection Group. Lineages are colored by their prioritization category in the March 2022 ranking. The colored bars indicate priority categories 1, 2, 3, and 4 in each month.

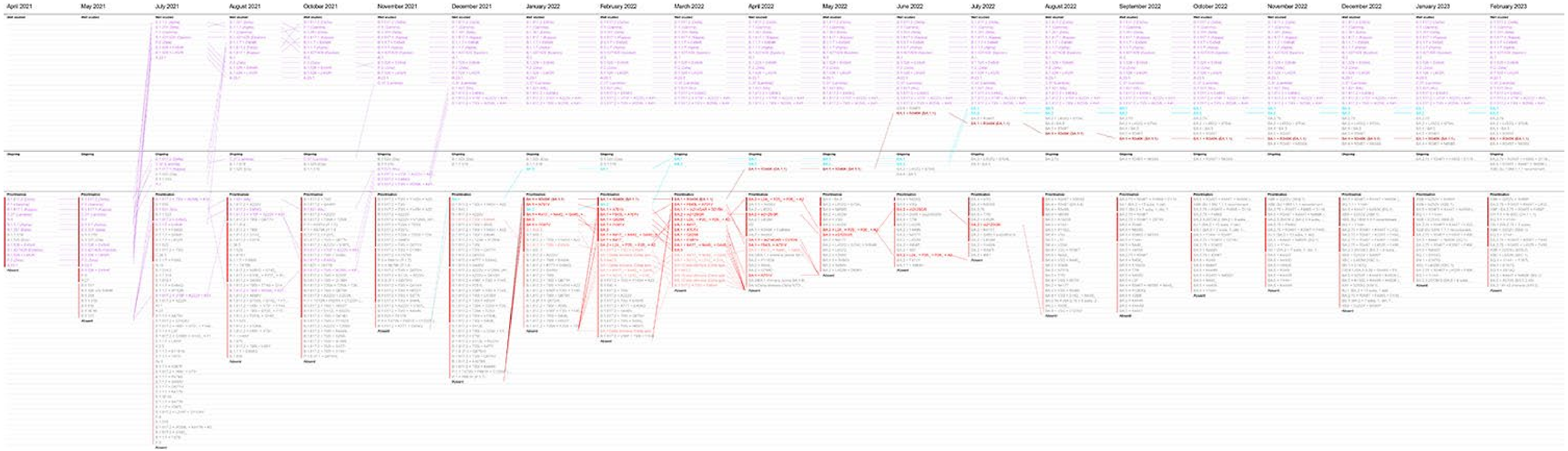

Appendix 3 Figure 16. Analogous to Appendix 2 Figure, for February 2022 prioritization, and showing all prioritizations. NIH SAVE Early Detection Prioritization of Variant rankings from 21 prioritizations between February 2023 and April 2021. The prioritizations are produced by taking the consensus of rankings provided by 9 subgroups which form the NIH SAVE Early Detection Group. Lineages are colored by their prioritization category in the February 2022 ranking. The colored bars indicate priority categories 1, 2, 3, and 4 in each month.

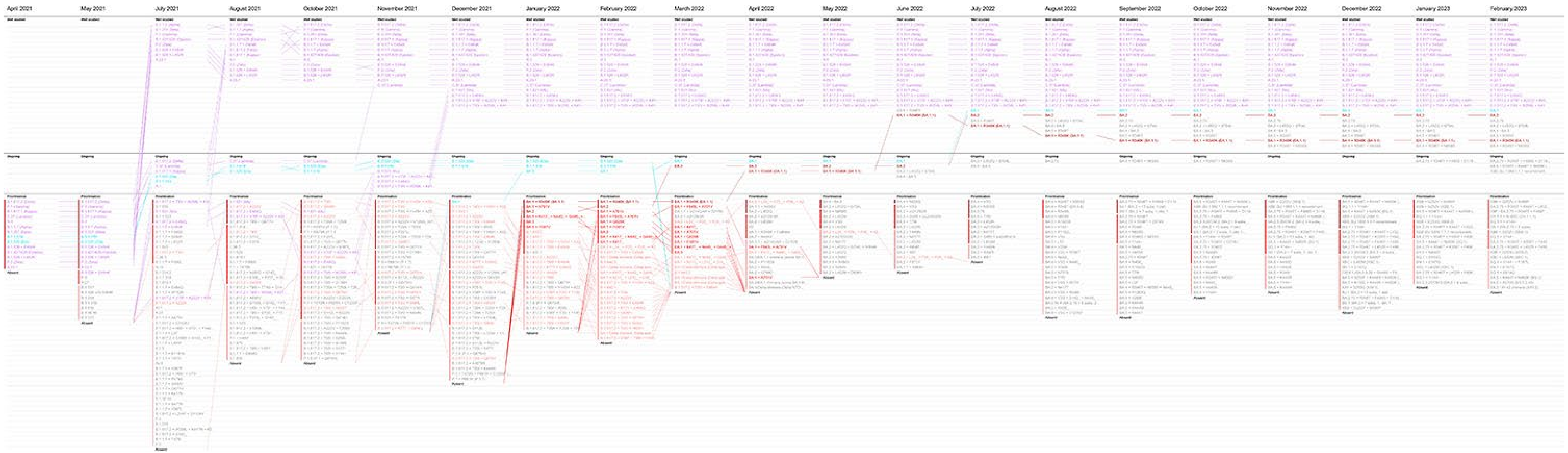

Appendix 3 Figure 17. Analogous to Appendix 2 Figure, for January 2022 prioritization, and showing all prioritizations. NIH SAVE Early Detection Prioritization of Variant rankings from 21 prioritizations between February 2023 and April 2021. The prioritizations are produced by taking the consensus of rankings provided by 9 subgroups which form the NIH SAVE Early Detection Group. Lineages are colored by their prioritization category in the January 2022 ranking. The colored bars indicate priority categories 1, 2, 3, and 4 in each month.

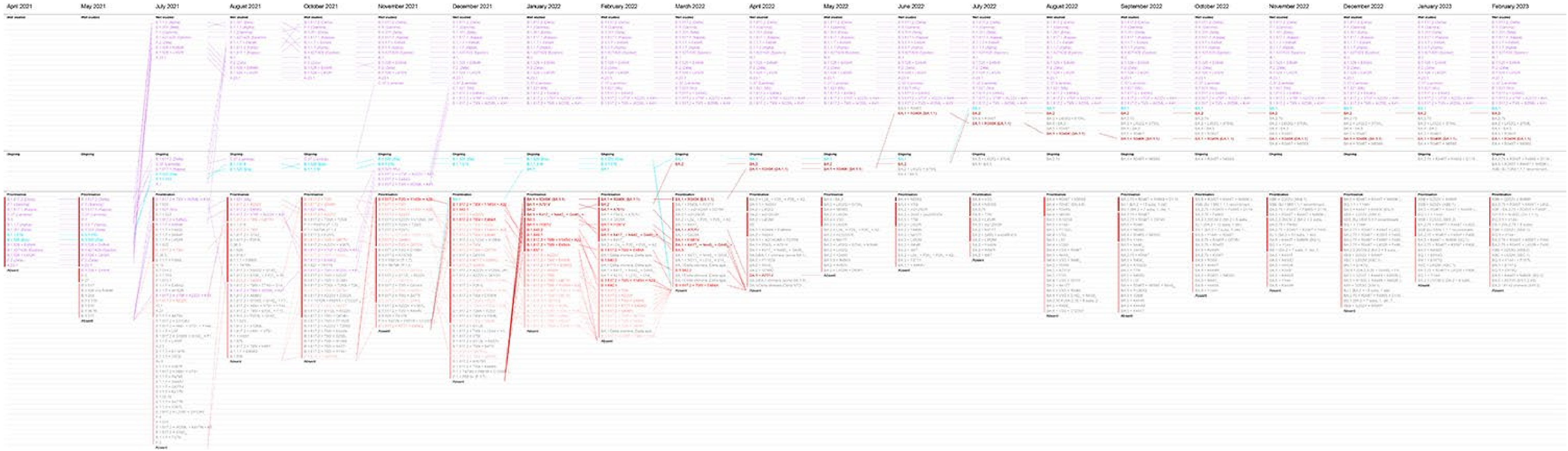

Appendix 3 Figure 18. Analogous to Appendix 2 Figure, for December 2021 prioritization, and showing all prioritizations. NIH SAVE Early Detection Prioritization of Variant rankings from 21 prioritizations between February 2023 and April 2021. The prioritizations are produced by taking the consensus of rankings provided by 9 subgroups which form the NIH SAVE Early Detection Group. Lineages are colored by their prioritization category in the December 2021 ranking. The colored bars indicate priority categories 1, 2, 3, and 4 in each month.

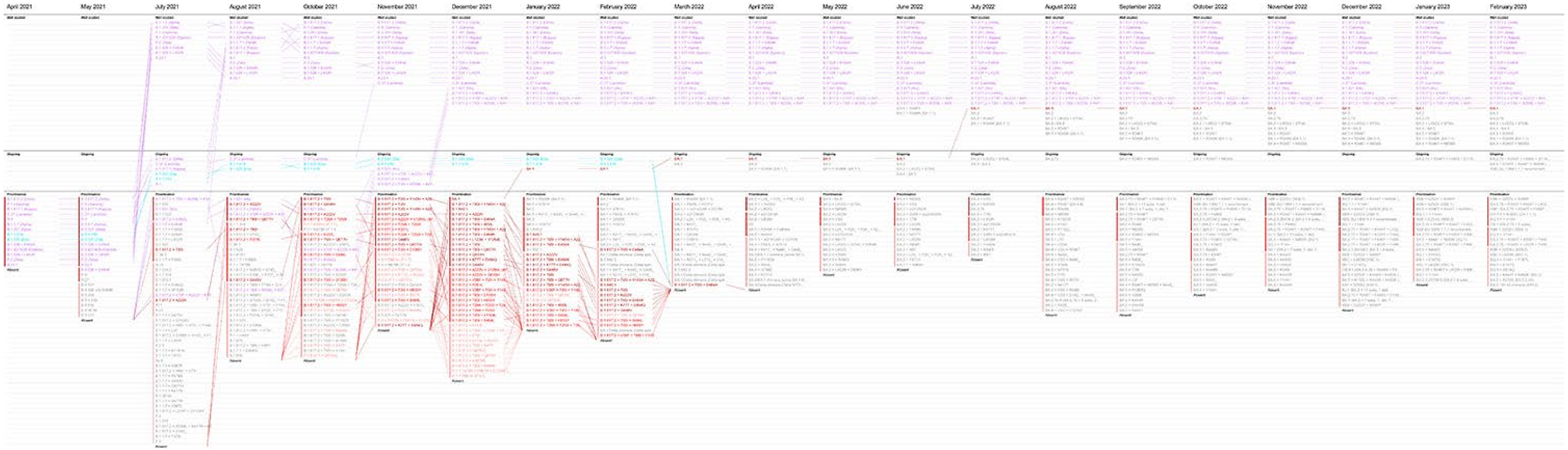

Appendix 3 Figure 19. Analogous to Appendix 2 Figure, for November 2021 prioritization, and showing all prioritizations. NIH SAVE Early Detection Prioritization of Variant rankings from 21 prioritizations between February 2023 and April 2021. The prioritizations are produced by taking the consensus of rankings provided by 9 subgroups which form the NIH SAVE Early Detection Group. Lineages are colored by their prioritization category in the November 2021 ranking. The colored bars indicate priority categories 1, 2, 3, and 4 in each month.

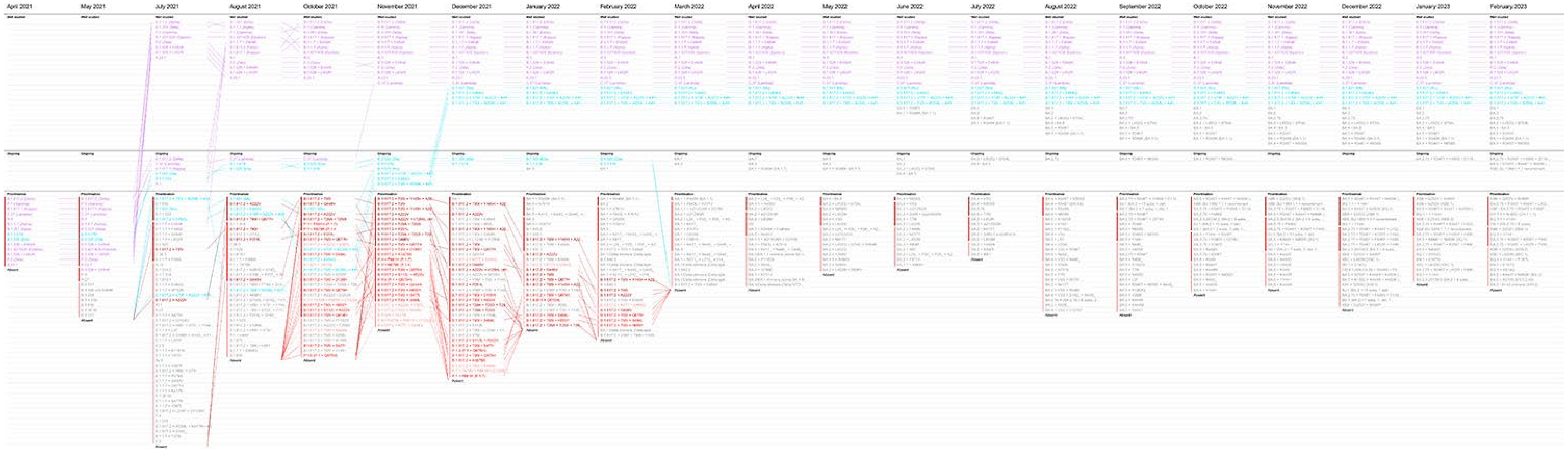

Appendix 3 Figure 20. Analogous to Appendix 2 Figure, for October 2021 prioritization, and showing all prioritizations. NIH SAVE Early Detection Prioritization of Variant rankings from 21 prioritizations between February 2023 and April 2021. The prioritizations are produced by taking the consensus of rankings provided by 9 subgroups which form the NIH SAVE Early Detection Group. Lineages are colored by their prioritization category in the October 2021 ranking. The colored bars indicate priority categories 1, 2, 3, and 4 in each month.

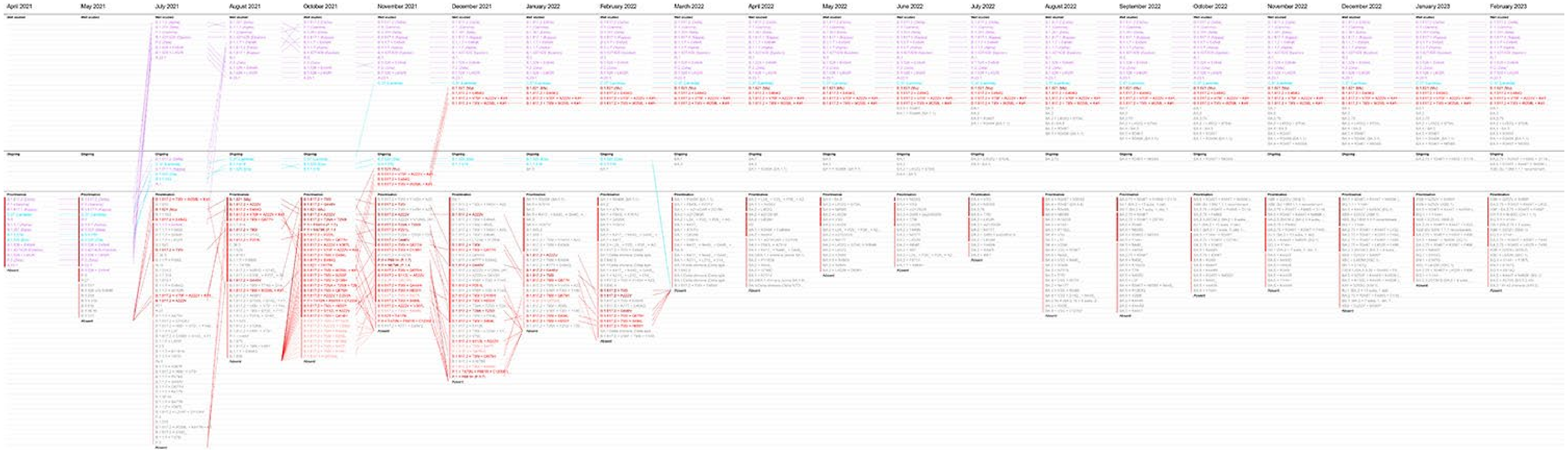

Appendix 3 Figure 21. Analogous to Appendix 2 Figure, for August 2021 prioritization, and showing all prioritizations. NIH SAVE Early Detection Prioritization of Variant rankings from 21 prioritizations between February 2023 and April 2021. The prioritizations are produced by taking the consensus of rankings provided by 9 subgroups which form the NIH SAVE Early Detection Group. Lineages are colored by their prioritization category in the August 2021 ranking. The colored bars indicate priority categories 1, 2, 3, and 4 in each month.

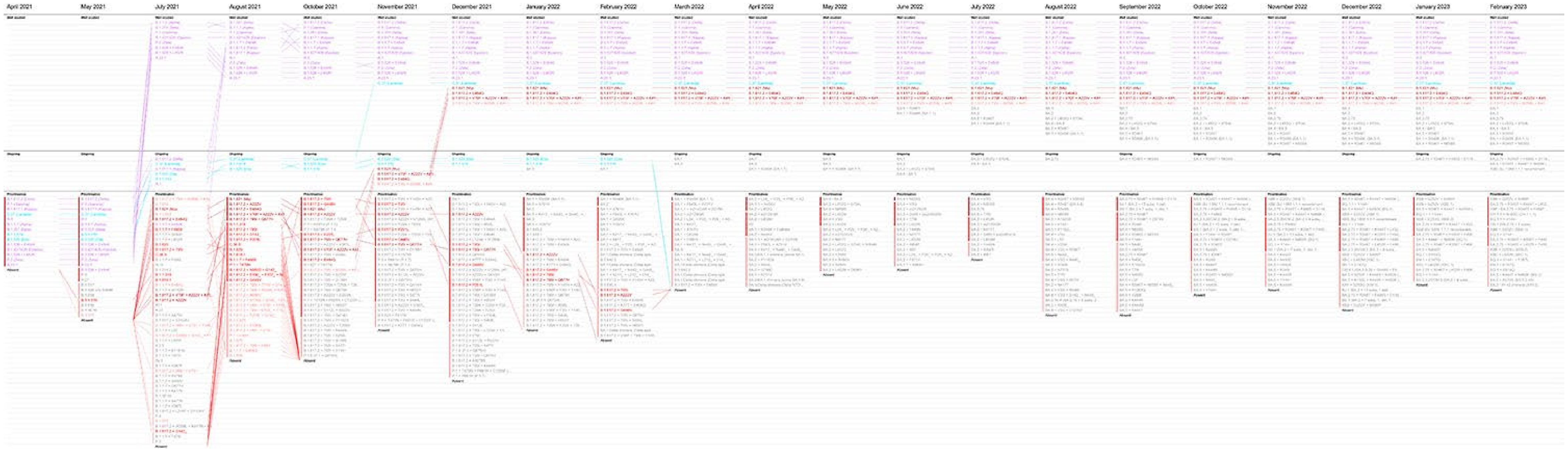

Appendix 3 Figure 22. Analogous to Appendix 2 Figure, for July 2021 prioritization, and showing all prioritizations. NIH SAVE Early Detection Prioritization of Variant rankings from 21 prioritizations between February 2023 and April 2021. The prioritizations are produced by taking the consensus of rankings provided by 9 subgroups which form the NIH SAVE Early Detection Group. Lineages are colored by their prioritization category in the July 2021 ranking. The colored bars indicate priority categories 1, 2, 3, and 4 in each month.

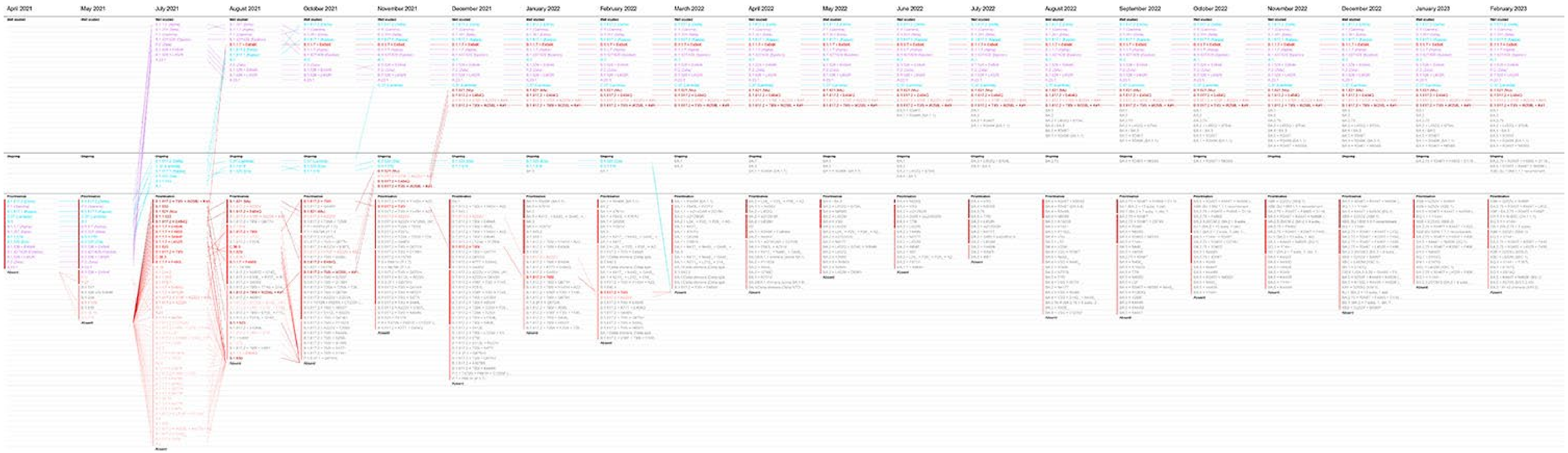

Appendix 3 Figure 23. Analogous to Appendix 2 Figure, for May 2021 prioritization, and showing all prioritizations. NIH SAVE Early Detection Prioritization of Variant rankings from 21 prioritizations between February 2023 and April 2021. The prioritizations are produced by taking the consensus of rankings provided by 9 subgroups which form the NIH SAVE Early Detection Group. Lineages are colored by their prioritization category in the May 2021 ranking. The colored bars indicate priority categories 1, 2, 3, and 4 in each month.

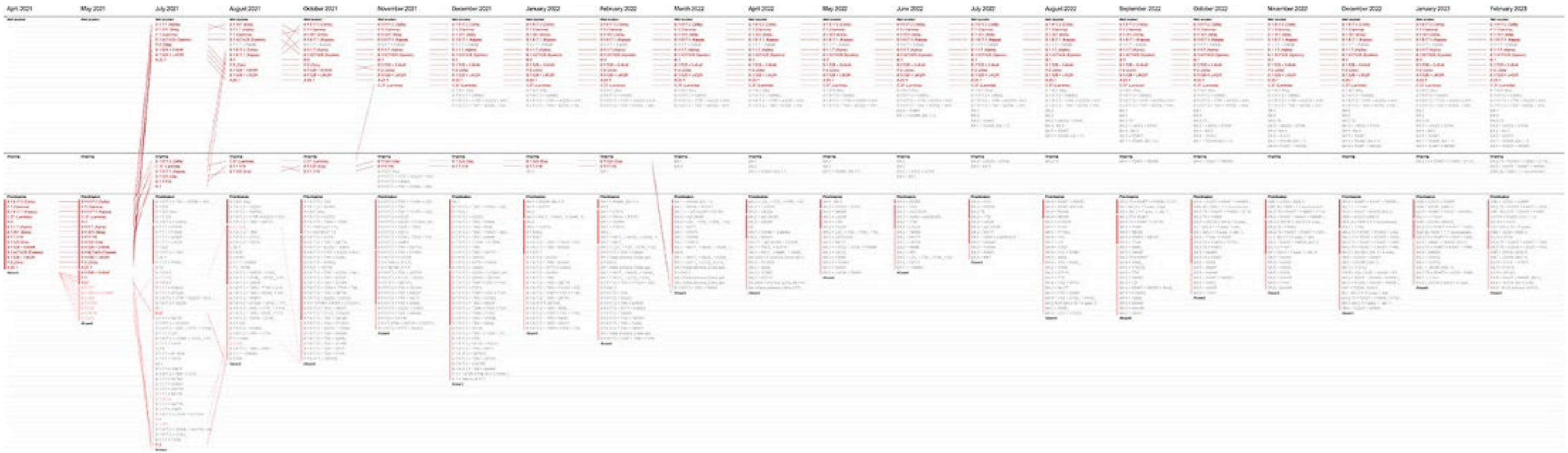

Appendix 3 Figure 24. Analogous to Appendix 2 Figure, for April 2021 prioritization, and showing all prioritizations. NIH SAVE Early Detection Prioritization of Variant rankings from 21 prioritizations between February 2023 and April 2021. The prioritizations are produced by taking the consensus of rankings provided by 9 subgroups which form the NIH SAVE Early Detection Group. Lineages are colored by their prioritization category in the April 2021 ranking. The colored bars indicate priority categories 1, 2, 3, and 4 in each month.
